# Food Entrainment, Arousal, and Motivation in the Neonatal Rabbit Pup

**DOI:** 10.3389/fnins.2021.636764

**Published:** 2021-03-17

**Authors:** Mario Caba, Michael N. Lehman, Mario Daniel Caba-Flores

**Affiliations:** ^1^Centro de Investigaciones Biomédicas, Universidad Veracruzana, Xalapa, Mexico; ^2^Brain Health Research Institute, Kent State University, Kent, OH, United States; ^3^Doctorado en Ciencias Biomédicas, CIB, Universidad Veracruzana, Xalapa, Mexico

**Keywords:** food entrainment, paraventricular nucleus, oxytocin, corticosterone, sympathetic system, parasympathetic system, reward, median preoptic nucleus

## Abstract

In the newborn rabbit, the light entrainable circadian system is immature and once a day nursing provides the primary timing cue for entrainment. In advance of the mother’s arrival, pups display food anticipatory activity (FAA), and metabolic and physiological parameters are synchronized to this daily event. Central structures in the brain are also entrained as indicated by expression of Fos and Per1 proteins, GFAP, a glial marker, and cytochrome oxidase activity. Under fasting conditions, several of these rhythmic parameters persist in the periphery and brain, including rhythms in the olfactory bulb (OB). Here we provide an overview of these physiological and neurobiological changes and focus on three issues, just beginning to be examined in the rabbit. First, we review evidence supporting roles for the organum vasculosum of lamina terminalis (OVLT) and median preoptic nucleus (MnPO) in homeostasis of fluid ingestion and the neural basis of arousal, the latter which also includes the role of the orexigenic system. Second, since FAA in association with the daily visit of the mother is an example of conditioned learning, we review evidence for changes in the corticolimbic system and identified nuclei in the amygdala and extended amygdala as part of the neural substrate responsible for FAA. Third, we review recent evidence supporting the role of oxytocinergic cells of the paraventricular hypothalamic nucleus (PVN) as a link to the autonomic system that underlies physiological events, which occur in preparation for the upcoming next daily meal. We conclude that the rabbit model has contributed to an overall understanding of food entrainment.

## Introduction

Mammals usually forage and consume food during their period of activity, which is controlled by the biological clock in the brain, the suprachiasmatic nucleus (SCN), with light serving as their main entraining signal (Finger et al., 2020). Thus, nocturnal rodents rest during the day but at evening increase their locomotor activity. However, when food is withheld and provided for a short period at a fixed time during the day, the normal activity pattern of animals shift from that controlled by the SCN and animals show now intense locomotor behavior, termed food anticipatory activity (FAA; Mistlberger, 1994) before food availability. In addition, a number of other physiological and neural parameters also are entrained. Early studies proposed that this phenomenon was controlled by an oscillatory system entrained by food, named the food-entrainable oscillator (Stephan, 2002). Now there is general agreement that FAA is controlled by a diffuse system of central and peripheral structures (Mistlberger, 2020) but the precise mechanism of their action it is not well understood. In contrast, rabbit pups in nature eat once a day for a few minutes. They have been recognized as a model of FAA, and offer an opportunity to study food entrainment with little manipulation. Here we first review what is known about the SCN in rabbit pups, including its responsiveness to light, as well as review evidence about peripheral parameters and central structures that are entrained by periodic food ingestion. Then we focus on three aspects of food entrainment that have been relatively unexplored in the rabbit. First, we focus on forebrain areas activated during arousal at the time of FAA, considering evidence supporting a role of the median preoptic nucleus (MnPO) in neural control of FAA and related peripheral events. Second, since periodic food ingestion implicates a conditioning process we also propose a neural substrate of motivational conditioning underlying FAA. Third, finally we address recent evidence in the rabbit pup regarding a population of oxytocinergic cells in the brain that may play a central role in coordinating mechanisms between central and peripheral structures in FAA. Finally, we compare results in the rabbit with those seen in other species and consider the usefulness of the rabbit pup for the study of food entrainment in an evolutionary context.

## Immature Light Entrainable Oscillator

Altricial mammals, such as the hamster and the rat, at postnatal day 1 (PD1) possess few or no retinal projections from the retinohypothalamic tract to the SCN (Speh and Moore, 1993). The rabbit pup is also altricial and spends all its time in a dark nest without receiving light. Tract tracing studies using Cholera toxin B subunit, revealed that, contrary to rodents, the SCN of the rabbit at PD1 receives a dense innervation of retinal projections with a predominantly contralateral pattern throughout the entire SCN (Juárez et al., 2013). A light pulse induces robust expression of the protein FOS, a marker of neural activity, in the SCN of the rabbit pup at PD1 although the adult pattern is not reached until PD19, after opening of the eyelids (Juárez et al., 2013). However, in this period before reaching the adult capacity to be entrained by light the neonatal rabbit exhibits a remarkable characteristic, it presents robust entrainment periodic to food.

## Food, a Strong Synchronizer of Locomotor Behavior

Rabbit pups are alone in the nest in darkness and spend most part of the time huddled with little movement (Jilge, 1993), which helps to maintain their core body temperature (Bautista et al., 2008). In nature (Lloyd and McCowan, 1968) and in laboratory conditions, the mother nurses her pups with circadian periodicity and pups show a sharp increase in locomotor behavior at around 3 h before daily suckling of milk (Caba and González-Mariscal, 2009; González-Mariscal et al., 2016). Nursing lasts around 5 min and immediately after locomotor behavior sharply decreases and thereafter pups exhibit low activity until expectancy of next nursing bout (Jilge, 1993, 1995; González-Mariscal et al., 2016). This behavioral pattern persists when pups remain un-nursed for 48 h and is evident starting around postnatal day 2 (Caba et al., 2008; Trejo-Muñoz et al., 2012).

## Physiological, Metabolic, and Hormonal Parameters Associated With FAA

Core body temperature increases 2–3 h prior to nursing which persists in un-nursed pups (Jilge et al., 2000; Trejo-Muñoz et al., 2012). Upon nursing stomach weight sharply increases and induces a sequential use of fuels, first glucose, then liver glycogen and finally free fatty acids in fasted subjects (Escobar et al., 2000; Morgado et al., 2008, 2010). We explored the patterns of the secretion of corticosterone (CORT) and ghrelin under these conditions. In contrast to rat pups (Levine, 2002), rabbit pups show a CORT rhythm entrained by nursing, which persist in un-nursed pups (Rovirosa et al., 2005; Morgado et al., 2008, 2010). With respect to ghrelin, 12 h after milk ingestion there is a sharp increase, which coincides with the emptying of most parts of the stomach (Morgado et al., 2008, 2010). This is interesting as ghrelin increases before meal ingestion in several species when subjects start to be hungry (Williams and Cummings, 2005); in addition, there is a premeal activation of ghrelin in oxyntic cells (LeSauter et al., 2009). However, ghrelin by itself it is not necessary for FAA in mice (Gunapala et al., 2011).

## Rhythms in Olfactory Bulb and Other Brain Structures Associated With FAA

The behavioral response of rabbit pups to the nipple’s mammary pheromone (2-methyl-but-2-enal; 2MB2) is highest during FAA (Schaal et al., 2003; Coureaud et al., 2004; Montigny et al., 2006), which suggests that changes also occur in the olfactory bulb (OB). Indeed, expression of FOS, by immunocytochemistry, used as neural marker of activity, shows rhythms entrained by the time of suckling of milk (Nolasco et al., 2012). At this time both Fos and cytochrome oxidase activity, a marker of metabolic activity (Wong-Riley, 1989), are highest and persist in fasted subjects (Nolasco et al., 2012; Olivo et al., 2014). Also Per1, an indicator of circadian oscillation, shows robust rhythms in the OB, entrained by nursing (Nolasco et al., 2012; Montúfar-Chaveznava et al., 2013). In addition, astrocytes in the OB also show daily changes in the length of radial processes and in expression of glial fibrillary acidic protein, which are associated to nursing time (Vázquez et al., 2020). It is necessary to explore the role of glia in the circadian rhythmicity in the OB. In contrast the SCN at this age is immature (Montúfar-Chaveznava et al., 2013). This suggests that the OB may be of major importance for the entrainment to daily nursing, and is also supported by evidence that the OB in adult rodents displays daily changes in sensitivity to odors (Amir et al., 1999; Granados-Fuentes et al., 2006), can be entrained by daily meals (Caba et al., 2014), and contains a circadian clock independent of the SCN (Granados-Fuentes et al., 2006). However, while bilateral destruction of the OB in the rabbit disrupts FAA (Navarrete et al., 2016) it has no or little effect on FAA in adult rats (Davidson et al., 2001).

Other brain areas have also been implicated in FAA in the rabbit pup. Similar to the OB, the dorsomedial hypothalamic nucleus (Caba et al., 2008) and the parabrachial nucleus (Juárez et al., 2012) are entrained, as indicated by PER1, reaching a peak 4–8 h after nursing, and their oscillations persist during fasting. In contrast the dorsal vagal complex (Juárez et al., 2012) only express FOS after nursing. Taken together, these results suggest that rabbit pups depend on multiple brain structures to entrain to daily food intake.

## Role of the Organum Vasculosum of Lamina Terminalis, Thirst and Arousal

**FIGURE 1:**
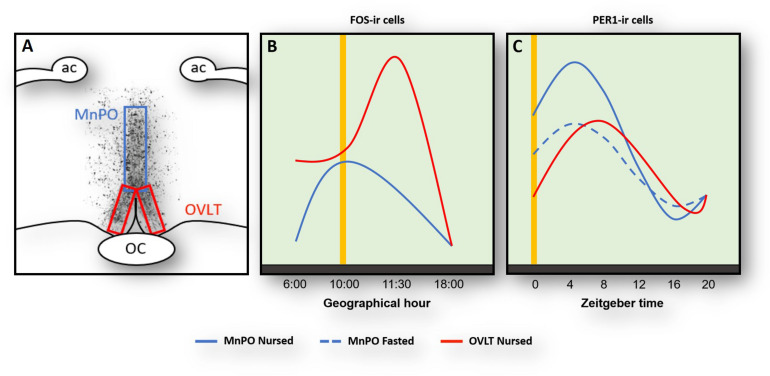
Activation of Fos- **(A,B)** and Per1**(C)**-ir cells in the median preoptic nucleus (MnPO) and in the organum vasculosum of lamina terminalis (OVLT) in relation to nursing (yellow vertical line). Panel **(A)** shows the location of the MnPO and OVLT. **(B)** At the time of nursing Fos increases in both OVLT (red line) and MnPO (blue) with a further increase 1.5 h after in the MnPO. **(C)** Per1 increases in both OVLT and MnPO 4–8 after nursing, but this rhythm persists in un-nursed pups only in the MnPO (dashed blue line). Modified from Moreno et al., 2013, 2014. ac, anterior commissure; OC, optic chiasma.

Rabbit pups ingest a large volume of milk at the time of suckling and do not drink additional fluids for the remaining 24 h. To better understand this continuous cycle of fluid balance in the brain we explored the role of the organum vasculosum of lamina terminalis (OVLT). This organ contains osmoreceptors and their destruction together with the adjacent MNPO significantly disrupts thirst and fluid balance (Johnson and Buggy, 1978; McKinley et al., 1999). In the rabbit pup, we saw a marked increase of FOS protein expression in the OVLT at 4 h before nursing (Figure 1) likely reflecting a signal of thirst. In support of this, pharmacological induction of thirst in rats produces a large increase of Fos in the OVLT (Thunhorst et al., 1998). Additionally, we also observed a large postprandial increase of Fos protein, which is consistent with the large volume of milk ingested (Figure 1), and 8 h. After nursing Fos reaches its lowest levels (Figure 1; Moreno et al., 2013) and stomach weight steadily decreases. At the time of next FAA, the stomach is almost empty (Morgado et al., 2008, 2010) and Fos increases again in the OVLT. In contrast, under fasting conditions, Fos levels remain high at all times (Moreno et al., 2013). In a further study we examined Per1 protein and found a clear rhythm with a peak 8 h after nursing indicating that this rhythm was entrained by this event; as in the case of Fos, no rhythm was observed in fasted pups (Figure 1). Overall, we conclude that the OVLT actively participates in the osmoregulatory control of milk ingestion and perhaps its activation before nursing contributes to the expression of FAA though connections with other brain areas, particularly the MnPO where thirst signals are integrated and in the cerebral cortex induce drinking behavior (McKinley et al., 2015).

## Medial Preoptic Area and Core Body Temperature

At the time of FAA, the core body temperature of rabbit pups also increases. Neurons in the preoptic area (POA) receive ascending peripheral thermosensory signals that are integrated in this nucleus and *via* projections then regulate the dorsomedial hypothalamic nucleus to promote thermogenesis (Morrison, 2016). In the rabbit we found an increase of Fos protein in the POA at the time of nursing coinciding with the increase in core body temperature at the same time. Lower values were found at other time points of the cycle (Moreno et al., 2013). However, no rhythm of Fos was observed in un-nursed pups, and also no rhythm in Per1 protein was observed in nursed and fasted pups (Moreno et al., 2014). In contrast the rhythm of body temperature persists in un-nursed pups (Jilge et al., 2000; Trejo-Muñoz et al., 2012). Thus the observed effect on Fos at the time of nursing in the POA seems not to be related to the rhythm of temperature.

## Role of the Median Preoptic Nucleus and the Orexinergic System in FAA

Immediately after FAA and milk ingestion, the rabbit pup’s activity sharply decreases and pups remain huddled with very little movement for around next 20 h. In considering this change from a heightened state of alertness to an almost quiescent state suggestive of sleep, we decided to explore regions in the POA that integrate information related to the control of the sleep/wake cycle. We centered our attention in the MnPO, which is an integrative center in the rostral wall of the third ventricle in the forebrain that plays a key role in the sleep/wake cycle (Suntsova et al., 2007; Sakai, 2011; McKinley et al., 2015). In nursed pups we found an anticipatory increase of Fos at the time of nursing and low levels before and after that event (Figure 1). A similar pattern, with a delayed increase 1.5 after nursing, was found in fasted pups (Moreno et al., 2013). In addition, we explored Per1 protein and found a clear rhythm in both nursed and fasted pups with higher values at the time and thereafter nursing and lowest levels 16 h after (Figure 1). Notably, in the same study we determined possible rhythms of Per1 in the OVLT, MPOA, and the MnPO and only in this latter structure did the rhythm persist in un-nursed pups (Moreno et al., 2014). MnPO activation seen as increases in Fos protein had previously been associated with sleep or sleep pressure (McKinley et al., 2015). As pups remains quiescent, perhaps sleeping, after FAA it is possible that this increase in Fos indicates sleep pressure. However, activation of this nucleus at the time of nursing could be related also to FAA. Electrophysiological studies revealed that MnPO contained similar proportion of neurons that showed increased discharge during either sleep or waking state (Sakai, 2011). The author of that study suggested that in contrast to the classical view that the MnPO plays a role only in sleep, this nucleus might modulate a differential role between sleep and wake states. This proposal is consistent with the observed persistence of Per1 rhythms. In support of this the MnPO send projections to the orexinergic cells in the perifornical area of the lateral hypothalamus to modulate sleep/awake states (Uschakov et al., 2007; Sakai, 2011; McKinley et al., 2015). These orexinergic cells are active at the time of FAA in mice (Mieda et al., 2004) in adult rats (Jiménez et al., 2013), and in the rabbit (Moreno et al., 2013). To our knowledge the MnPO has not been analyzed in relation to food entrainment in other species and this is an area worthy of further study.

## Motivation and the Extended Amygdala in FAA

In the adult rat, FAA shows features that implicate a process of conditioned learning (Silver et al., 2011). In order to explore brain mechanisms associated with conditioned learning of FAA, we analyzed several nuclei of the amygdala and the extended amygdala related to alertness and emotional arousal by using CO histochemistry (Olivo et al., 2017). During the period of FAA, we found activation in the basolateral, medial and central nuclei of the amygdala, bed nucleus of the terminalis, lateral septum, and nucleus accumbens core. This is interesting as the basolateral amygdala mediates the acquisition of associative learning and together with other nuclei of the extended amygdala participates in the emotional processing of stimuli (Davis and Whalen, 2001; Namburi et al., 2015). Also, in food-restricted rats, the basolateral amygdala, as well as other regions of the corticolimbic system, is entrained as indicated by Fos and Per1 proteins (Angeles-Castellanos et al., 2007). After food ingestion in the rabbit there was an increase in metabolic activity in the nucleus accumbens shell, caudate, putamen and cortical amygdala (Olivo et al., 2017), which further support a functional role in FAA for the circuit of food reward (Morales and Berridge, 2020). Overall, these results indicate a neural substrate for the conditioned learning in subjects that is induced by the nursing event and suggests that rabbit pups are motivationally aroused in expectation of receiving food.

## Oxytocin and a Central and Peripheral Network in Food Entrainment

**FIGURE 2:**
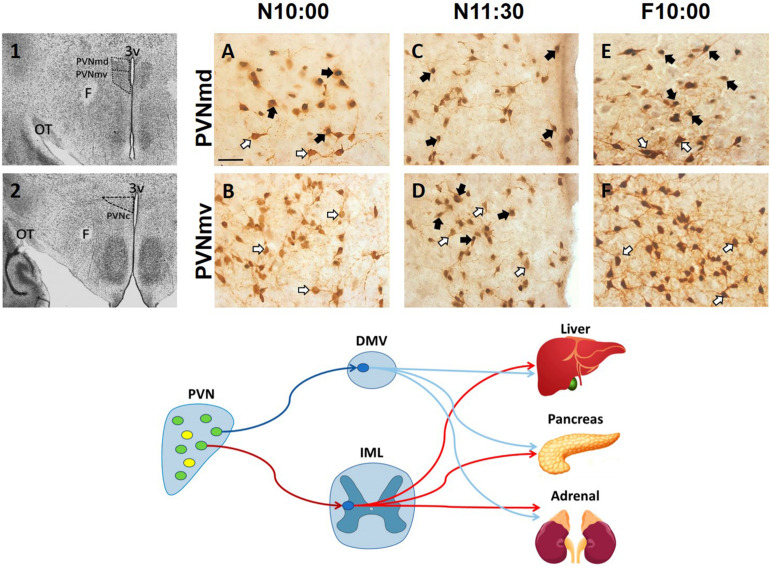
Activation of oxytocinergic cells in the paraventricular hypothalamic nucleus (PVN) that coincides with food anticipatory activity in rabbit pups. 1, 2: Photomicrographs showing the location of the dorsal (PVNmd) and ventral (PVNmv) portions of the main body of the PVN **(A)** and their caudal portion (PVNc) **(B)**. A-F: Expression of oxytocin (white arrows) and double-labeled oxytocin and Fos (black arrows)-ir cells in the PVNmd **(A,C,E)** and PVNmv **(B,D,F)** just before nursing at 10:00 am (N10:00), 1.5 h after (N11:30) and in fasted subjects at the time of the previous scheduled nursing (F10:00). Note the increase in Fos/OT-ir cells before nursing **(A)** that persist in fasted subjects **(E)** only in the PVNmd. In contrast, the PVNmv only shows an increase in FOS/OT-ir cells after suckling of milk **(D)**. OT, optic tract. Modified from Caba et al. (2020). Bottom panel. Schematic of non-OT cells (yellow) and interaction of PVN OT (green) pre-autonomic sympathetic (red) and parasympathetic (blue) neurons that project to the preganglionic sympathetic system (red) in the intermediolateral (IML) column of the spinal cord, or to the preganglionic parasympathetic system (blue) of the dorsal motor nucleus of the vagus (DMV) in the medulla, that control the neural outflow to peripheral organs. Adapted from Buijs et al. (2001, 2003).

After food ingestion there is an oxytocin (OT) release to the periphery and in several brain regions (Swanson and Kuypers, 1980; Olson et al., 1991; Verbalis et al., 1996; Spetter and Hallschmid, 2017), and OT projections to the brainstem are thought to be critical in a circuit underlying feeding and satiety (rev Swanson and Kuypers, 1980; McCormack et al., 2020). In the rabbit pup, milk ingestion induces an activation of OT neurons in both the SON and paraventricular hypothalamic nucleus (PVN) (Caba et al., 2003; Morgado et al., 2011). Peripheral OT plays a role in energy intake and expenditure processes including gastric emptying and distention, carbohydrate and lipid intake, fat oxidation, insulin secretion and glucose homeostasis (revs. Spetter and Hallschmid, 2017; McCormack et al., 2020). However, in the main body of the PVN, specifically in its dorsal and ventral portion and in its caudal region, we found an activation of OT cells *before* milk ingestion in the rabbit (Caba et al., 2020). In Figure 2, we show this effect in the two subregions of the main body of the PVN. This result suggests a differential activation of OT neurons in this nucleus related to preparatory actions for the upcoming meal. In the rat under food restriction, and in the rabbit pup before their daily period of milk intake, there is an increase in corticosterone, free fatty acids and glucagon indicating a catabolic state, and in parallel there is a decrease in glycogen and insulin (Díaz-Muñoz et al., 2000; Escobar et al., 2000; Morgado et al., 2008, 2010). We proposed (Caba et al., 2020) that the subpopulation of activated neurons in the PVN are related to these peripheral effects through identified preganglionic OT cells from the sympathetic and parasympathetic system that project from the PVN to the liver, pancreas, and adrenals (Figure 1, bottom panel; Buijs et al., 2001, 2003). It seems likely that this activation is associated with both central and peripheral roles of these cells in the context of food entrainment. Future studies need to explore the differential activation of these OT cells and their receptors to projected areas in order to determine their physiological importance for FAA.

## Conclusion

Although animals usually forage and eat during their period of activity, food is usually only available at a specific time either during the active or rest phase. In this respect, FAA represents an adaptive strategy in response to a limited, ecological resource. Although this phenomenon had been studied mainly in a few species of mammals, it had also been described in bees (rev. in Antle and Silver, 2009), and under laboratory conditions in zebrafish *Danio rerio* and in cavefish *Phreatichthys andruzzii* (Cavallari et al., 2011). Cavefish have evolved to live in darkness and although they express rhythmic clock genes they do not respond to light/dark cycles. On the other hand, under conditions of food restriction, cavefish show an increase in locomotor behavior before food availability indicative of FAA, as well as robust circadian rhythms of clock genes (Cavallari et al., 2011). This suggests that the ability for food entrainment is preserved across diverse taxa. Finally, it is evident that food is more important for survival than light. The rabbit pup presents an extraordinary opportunity to study FAA with little manipulation because this species shows this important evolutionary strategy only during the first 2 weeks of life before they open their eyes and start to be entrained by light.

## Author Contributions

MC, MNL, and MDC-F conceived and wrote the manuscript. All authors contributed to the article and approved the submitted version.

## Conflict of Interest

The authors declare that the research was conducted in the absence of any commercial or financial relationships that could be construed as a potential conflict of interest.
